# Identification of consistent post-translational regulatory triplets related to oncogenic and tumour suppressive modulators in childhood acute lymphoblastic leukemia

**DOI:** 10.7717/peerj.11803

**Published:** 2021-07-14

**Authors:** YongKiat Wee, Yining Liu, Min Zhao

**Affiliations:** 1 School of Science and Engineering, University of the Sunshine Coast, Sunshine Coast, QLD, Australia; 2 The School of Public Health, Institute for Chemical Carcinogenesis, Guangzhou Medical University, Guangzhou, China

**Keywords:** Childhood leukaemia, Oncogene, Tumour suppressor gene, Integrative network analysis, Gene regulatory

## Abstract

**Background:**

Acute lymphoblastic leukemia (ALL) is the most common type of childhood cancer. It can be caused by mutations that turn on oncogenes or turn off tumour suppressor genes. For instance, changes in certain genes including Rb and p53 are common in ALL cells. Oncogenes and TSGs may serve as a modulator gene to regulate the gene expression level via their respective target genes. To investigate the regulatory relationship between oncogenes, tumour suppressor genes and transcription factors at the post translational level in childhood ALL, we performed an integrative network analysis on the gene regulation in the post-translational level for childhood ALL based on many publicly available cancer gene expression data including TARGET and GEO database.

**Methods:**

We collected 259 childhood ALL-related genes from the latest online leukemia database, Leukemia Gene Literature Database. These 259 genes were selected from a comprehensive systematic literature with experimental evidences. The identified and curated genes were also associated with patient survival cases and we incorporated this pediatric ALL-related gene list into our analysis. We extracted the known human TFs from the TRRUST database. Among 259 childhood ALL-related genes, 101 unique regulators were mapped to the list of oncogene and tumour suppressor genes (TSGs) from the ONGene and the TSGene databases, and these included 74 TSGs, 62 oncogenes and 46 TF genes.

**Results:**

The resulted regulation was presented as a hierarchical regulatory network with transcription factors (TFs) as intermediate regulators connecting the top modulators (oncogene and TSGs) to the common target genes. Cross-validation was applied to the results from the TARGET dataset by identifying the consistent regulatory motifs based on three independent ALL expression datasets. A three-layer regulatory network of consistent positive modulators in childhood ALL was constructed in which 74 modulators (40 oncogenes, 34 TSGs) are considered as the most important regulators. The middle layer and the bottom layer contain 34 TFs and 176 target genes, respectively. Oncogenes mostly participated in positive regulation of gene expression and the transcription process of RNA II polymerase, while TSGs were mainly involved in the negative regulation of gene expression. In addition, the oncogene-specific targets were enriched with regulators of the MAPK cascade while tumour suppressor-specific targets were associated with cell death.

**Conclusion:**

The results revealed that oncogenes and TSGs possess a different functional regulatory pattern with regard to not only their biological functions but also their specific target genes in childhood ALL cancer progression. Taken together, our findings could contribute to a better understanding of the important regulatory mechanisms and this method could be used to analyse the targeted genes at the post-translational level in childhood ALL through integrative network analysis.

## Introduction

Cancer is caused by genetic mutations that can lead to uncontrolled cell growth and cell proliferation (Evan & Vousden, 2001). These mutated genes commonly play an important role in cancer development including cell death and cell proliferation (Evan & Vousden, 2001). There are two major categories of genes that contribute to cancer development in opposite ways. The first category is oncogenes that promote cell growth through gain of function mutations while the second category is tumour suppressor genes (TSGs) that inhibit apoptosis, slow down cell division and repair DNA mistakes that have arisen through loss of function mutations (Croce, 2008; Levine & Puzio-Kuter, 2010). The increased level in the oncogenic signalling pathway of the oncogenes can cause the imbalance of the apoptosis-inducing stresses signalling and as well as hyperproliferation that can induce DNA damage process (Hanahan & Weinberg, 2011). When the activation of the oncogenes is triggered by genetic alterations, the transforming activity will be increased by changing the structure of the encoded protein. Oncogenes are known as dominant genes including *RAS* and *MYC* in which they can trigger the overexpression of the angiogenic factors within the tumour cells. The occurrence of oncogenic mutations can alter the oncogenic signalling pathways, such as the diverse effects of the prominent oncogenes—mutated *RAS* and overexpressed *MYC* on the different hallmark capabilities (e.g. cell death, survival, cell growth and angiogenesis) (Hanahan & Weinberg, 2000).

In addition to the positive growth-stimulatory signals in the cancer hallmark capabilities, cancer cells must also be able to evade the powerful biological responses which negatively control the cell proliferation signalling pathways; many responses depend on the activity of TSGs (Hanahan & Weinberg, 2000). It encodes a protein which inhibits cell transformation and growth of cancerous cells. The two TSGs that encode the RB (retinoblastoma-associated) and TP53 proteins; they serve as a control in the cellular regulatory pathways by influencing the proliferation decisions, or alternatively, activate the cell death programs (Hanahan & Weinberg, 2011). Most notable is a DNA-damage sensor that operates via the *TP53* tumour suppressor (Junttila & Evan, 2009); *TP53* activates cell death by upregulating the expression level of the Noxa proteins, doing so in response to substantial levels of DNA breaks and other chromosomal abnormalities. Inactivation of tumour suppressor genes can also be acquired through epigenetic mechanisms such as DNA methylation and histone modifications (Berdasco & Esteller, 2010), some clonal expansions may be altered by non-mutational changes affecting the regulation of gene expression.

Over the past decade, oncogenes and TSGs have been categorized based on their functions in transformation events including cell death and cell proliferation (Croce, 2008). However, the molecular mechanisms underlying the regulation of oncogenes and TSGs are unclear, particularly at the post translational cellular levels. The products of oncogenes and TSGs can be divided into several groups, one of which contains the transcription factors (TFs). DNA-binding transcription factors possess an important role in gene regulation and their biological activities are often regulated by different molecules at the post-translational modification stage (Todeschini, Georges & Veitia, 2014). Previous study showed that oncogenes act as the post translational modulators which affect the activities of TFs (Wang et al., 2009; Zhao, Sun & Zhao, 2012). Most of the oncogenes and TSGs are not classified as transcription factors and it remains challenging to identify the relationship of the oncogenes and TSGs with TFs binding associates to the regulation of transcription. This is because these genes do not have the direct impact on the gene regulation. As a result, the oncogenes and TSGs may be regulated indirectly by modulation of the TFs at the post-translational level. The interrelation between key regulators of early T cell development and ALL oncogenic signals is best represented by the role of NOTCH1, an important T cell fate specification development factor, that is triggered by oncogenic gain-of-function mutations in over 60% of T-ALLs cases (Weng et al., 2004). Activating mutations in NOTCH1 normally happen with loss of the cyclin-dependent kinase inhibitor 2A (CDKN2A) locus (Kataoka et al., 2018), that eventually induce chromosomal rearrangements and causing in the abnormal expression of T cell-specific transcription factors which can operate as oncogenes. Another key genetic events in ALL are inactivation of tumour suppressor genes. The main reported tumour suppressor genes centre around the genetic events are p53 and Rb. Both of them play an essential regulatory role during G1 to S transition in the cell cycle. The Rb tumour suppressor gene is infrequently structurally mutated, but some of the ALL cases have seen to be losing expression of this key protein. Particularly, mutations of one of these genes p53 and Rb genes turn up to avert the need for inactivation of other genes in the regulatory pathway (Hatta & Koeffler, 2002). A novel tumour suppressor gene on chromosome 6q may also possess a vital role in the pathogenesis of ALL. As a result, tumour suppressor genes are frequently mutated in ALL and their mutation is probably the driving force for the transition from chronic to acute leukemia (Hatta & Koeffler, 2002).

The occurrence rates of childhood and adult cancers have increased in the recent years (Scollon et al., 2017). Acute lymphoblastic leukemia (ALL) is one of the common childhood malignancies and represents one-third of all cancer diagnoses in children (Pui et al., 2012). Genetic alterations of oncogenes and TSGs play a significant role in ALL. Activation of oncogenic transcription factors from the rearrangement of T cell receptors is one of the key features in the development of ALL such as *TLX3* and *IKZF* gene. Furthermore, the many genetic pathways involved in ALL progression, such as haematopoiesis and, prompts questions about how the gene regulatory network integrates signal to respond to cell death, proliferation and activation of leucocyte and lymphocytes (Li et al., 2017). We hypothesized that a systematic integration of TFs with their corresponding potential modulators (oncogenes and TSGs) may provide a new perspective to explore the gene regulatory network in paediatric ALL. Moreover, this approach will also provide clues to deciphering the gene expression regulatory networks that are associated with several important biological processes including cell death and cell proliferation.

## Methods

### Collection of childhood ALL-related genes and gene expression data sets

**Figure 1:**
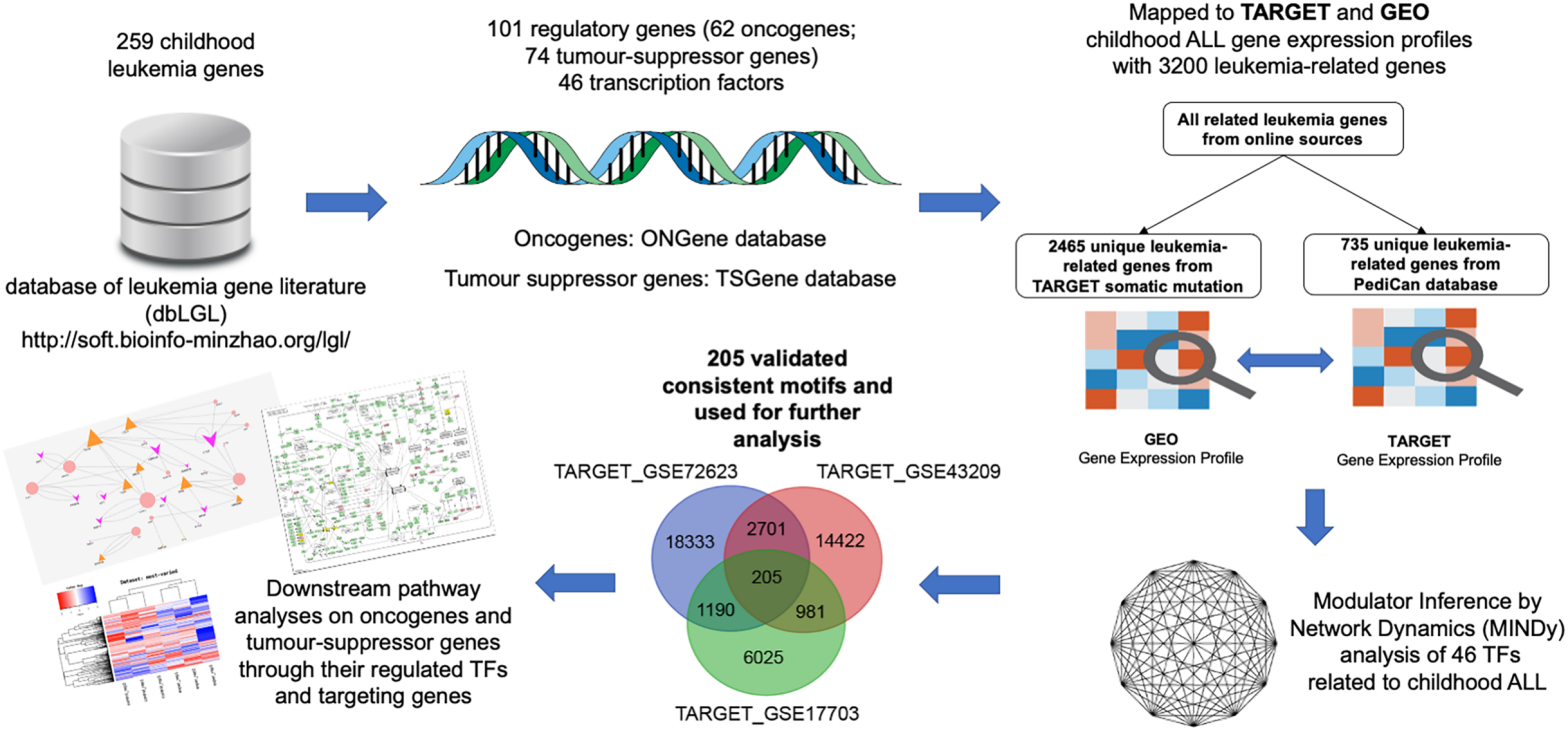
Flow chart of regulatory network analysis. This figure illustrates the computational strategy of regulatory network construction and the discovery of significant downstream pathways regulated by oncogenes and TSGs. Our computational approach involves four steps: (1) Collecting and downloading all the childhood ALL-related genes from the dbLGL database (http://soft.bioinfo-minzhao.org/lgl/); (2) incorporating the TARGET gene expression profile to build a regulatory network with leukemia-related oncogenes, TSGs, transcription factors and target genes; (3) comparing the regulatory results based on different childhood ALL gene expression profiles—TARGET and GEO for the identification of consistent motifs; and (4) investigating the important downstream pathways and identifying the relationship of oncogenes, TSGs and their corresponding modulating TFs in a particular biological process. Modulator Inference by Network Dynamics (MINDy) was applied to determine the vital modulators of transcription factors based on the gene expression profiles at the post-translational level.

We collected 259 childhood ALL-related genes from the latest online leukemia database, Leukemia Gene Literature Database (Liu et al., 2018). These 259 genes were selected from a comprehensive systematic literature review of genes with experimental evidences. The identified and curated genes were also associated with patient survival cases and we incorporated this paediatric ALL-related gene list into our analysis.We extracted the known human TFs from the TRRUST database (Han et al., 2015). Among 259 childhood ALL-related genes, 101 unique regulators were mapped to the list of oncogene and TSGs from the ONGene (Liu, Sun & Zhao, 2017 and the TSGene (Zhao et al., 2016) databases, and these included 74 TSGs, 62 oncogenes and 46 TF genes in the constructed hierarchical network as shown in Fig. 1.

The TARGET Acute Lymphoblastic Leukemia projects annotate inclusive molecular classification to identify the genetic alterations which are involved in childhood cancers progression (Liu et al., 2017). Using the completed ALL Pilot Phase (Phase I) data, we downloaded all the mRNA expression data from the TARGET public database (https://ocg.cancer.gov/programs/target/data-matrix). In the dataset, there are 180 childhood ALL samples that were examined using Affymetrix U133 Plus 2.0 Array platform. The affymetrix controls was applied to all the arrays as stated in their protocol (https://ocg.cancer.gov/programs/target/target-methods#396). The final gene expression data downloaded from TARGET database was then formatted as a matrix; each row represents a gene and each column represents a sample.

### Construction of a hierarchical gene regulatory network based on childhood ALL TARGET expression profiles

To find the correlation of the ALL-related genes and construct the ALL gene regulatory network, we integrated a total of 3,200 unique leukemia-associated genes from different sources, including 2,465 unique leukemia genes from TARGET childhood somatic mutations and 735 leukemia-related genes from the latest online gene resource for paediatric cancers, PediCan database (Zhao et al., 2015). We then extracted the ALL expression profiles for the 3200 genes from the 12,100 gene expression profiles in TARGET. We utilized the software MINDy (Modulator Inference by Network Dynamics) to infer the regulatory relationship between oncogenes, TSGs and TF genes. MINDy applied the conditional mutual information to determine the potential modulators that regulate the activity of a TF based on the inputted gene expression profiles (Wang et al., 2009). Four types of input data were required in MINDy: a gene expression profile; a TF gene list of interest; a list of putative modulator genes; and a list of potential TF targets. The first input data for the analysis was the gene expression matrix which comprised of 3,200 genes from the 180 childhood ALL TARGET samples. The TF gene list of interest included the overlapping 46 TF genes with the 259 childhood ALL-related genes. The modulators contained matched 62 oncogenes and 74 TSGs while the remaining genes in the 3,200 list were considered as the potential target genes. We used a default cut-off score of 0.1 in MINDy to determine the positive modulator where the modulator enhances the transcription factor and target interaction. As a result, the outputted gene list contained 44 TFs, 101 modulators (44 oncogenes and 57 TSGs) and 3,108 target genes which were used for further analysis.

### Independent validation of regulatory motifs based on different gene expression profiles (TARGET and GEO)

In order to identify the consistent motif in childhood ALL, we conducted MINDy analyses by using the similar computational approach with three different gene expression profiles. We collected 170 paediatric ALL patients from three cohorts (GSE17703—100 samples, GSE43209—40 samples and GSE72623—30 samples) downloaded from GEO database. We performed the MINDy analysis for each GEO gene expression profile separately. A genome-wide microarray analysis of a total of 100 Chinese paediatric ALL samples was carried in the GSE17703 dataset (Zou et al., 2012). While for the GSE72623 dataset, Palmi et al. analysed the gene expression profile of 212 ALL paediatric patients enrolled in AIEOP-BFM ALL2000 study in Italian and German centre (Palmi et al., 2016). Each MINDy results from three GEO datasets were compared with TARGET MINDy result. For this purpose, we applied the same previous computational pipeline on these GEO datasets and the similar default cut-off MINDy score (greater than 0.1) was determined to ensure the consistency between results. Based on the comparison of both MINDy results from two different datasets (TARGET and GEO), we identified 205 consistent motifs with the identical 34 transcription factors, 74 modulators (40 oncogenes and 34 TSGs) and 176 target genes (Table S1). By overlapping both MINDy results, we constructed a gene regulatory network of the identified constant motifs. We used the gene list from Affymetrix U133 Plus Array as the background of our gene ontology overrepresentation analysis.

### Gene network topological analyses

To measure the topology of the constructed gene regulatory network, we utilised Cytoscape (Shannon et al., 2003) for calculating the degree, closeness centrality and betweenness centrality of our network. While for the background of our topological analysis, we used the random networks for the comparison of oncogenes and tumour suppressor genes. The degree is the number of connections of a node or regulator where it calculates the connections of each of the regulators in the gene regulatory network (Barabasi & Oltvai, 2004). Closeness centrality, also known as shortest-path distance, indicates the average of the shortest path between a certain node and other node. For instance, the node here represenst the regulator of this analysis (Barabasi & Oltvai, 2004) while betweenness specifies the percentage of how often a node is identified on the shortest oath from all other shortest paths (Barabasi & Oltvai, 2004). The *p*-value from the closeness and betweenness centrality was obtained from the list of genes in the Network Analyzer from the calculated metrices based on the directed network.

### Constructing subnetworks on cell death, cell proliferation, haematopoiesis and activation of leucocytes and lymphocytes

To provide a more in-depth understanding of the specific biological modules in our gene regulatory network, we focused on the four functional processes were identified in our enriched regulatory network which have been reported in childhood ALL cancer progression. These processes were: cell death, cell proliferation, haematopoiesis, and activation of leucocyte and lymphocytes (Fridman & Lowe, 2003; Ozaki & Nakagawara, 2011). We gathered four biological term lists on cell death, cell proliferation, haematopoiesis and activation of leucocytes and lymphocytes using the Gene Ontology terms from the Toppfun online analysis tool (Chen et al., 2009). Lastly, we collected 28 gene lists for cell death, 38 for cell proliferation, 20 for haematopoiesis and 25 for activation of leucocyte and lymphocytes in our hierarchical regulatory network based on the functional term curations (Table S2).

Most of the genes collected from the four functional sets could be mapped to the top and bottom layers in the regulatory network. In order to derive a subnetwork from our regulatory network, the collected genes were firstly mapped to the bottom layer, then we identified the TFs which have connections to the target genes at the middle layer, and lastly, we determined those oncogenes and TSGs at the top layer which have the connections with these TFs. Overall, we acquired four subnetworks by implementing this approach.

### Construction of TFs and target gene profiling of oncogenes and TSGs

To examine the downstream childhood-related target genes of oncogenes and TSGs from the identified consistent motifs, we investigated the presence of target genes. Then we established a target profile for each corresponding oncogenes and TSGs in our constructed hierarchical gene regulatory network. If a regulatory relationship was identified in each oncogene or tumour suppressor gene, it was given a value of one; all others were scored zero. The dot plot was constructed using ggplot2 in R. Therefore, our target profile contained 238 entries with 0 or 1 for each oncogene or tumour suppressor gene.

## Results

### Overview of the computational pipeline to construct a hierarchical regulatory network

To examine the network interactions for post-regulatory mechanisms of oncogenes and TSGs modulated by TFs, we developed a computational framework to generate a combinatory gene regulatory network of oncogenes, TSGs, their respective modulating transcription factors and regulating joint target genes. The framework began with a gene list of paediatric leukemia-related genes, oncogenes, TSGs and transcription factors that compiled from different sources (see “Methods”). A total 259 literature-based ALL-related genes were used for investigation in ourcomputational framework. Next, a total of 3,200 genes involved in leukemia were extracted from all the gene expression profiles. Those from the prepared gene list and expression file were served as an input file in MINDy to interrogate the roles of oncogenes and TSGs as modulator of transcription factors at the post-translational level in childhood ALL. The resulted file contains a list of gene regulatory triplets which consisted of an oncogene and tumour suppresser gene as modulator (top layer), TF (middle layer) and joint target gene of modulator and TF (bottom layer). We converted the results into a motif format with four columns (transcription factors, modulators, target genes, MINDy score). To perform the cross-validation analyses of the identified motifs, we compared the MINDy results from TARGET with the results from three different GEO data sets. As a result, a final three-layer regulatory network was constructed based on the validated consistent positive motifs with default MINDy score greater than 0.1. The final network comprised of 205 validated consistent motifs with identical 34 TFs, 74 modulators (40 oncogenes and 34 TSGs) and 176 target genes.

### A paediatric ALL-specific regulatory gene network modulated by validated consistent oncogenes and TSGs

**Figure 2:**
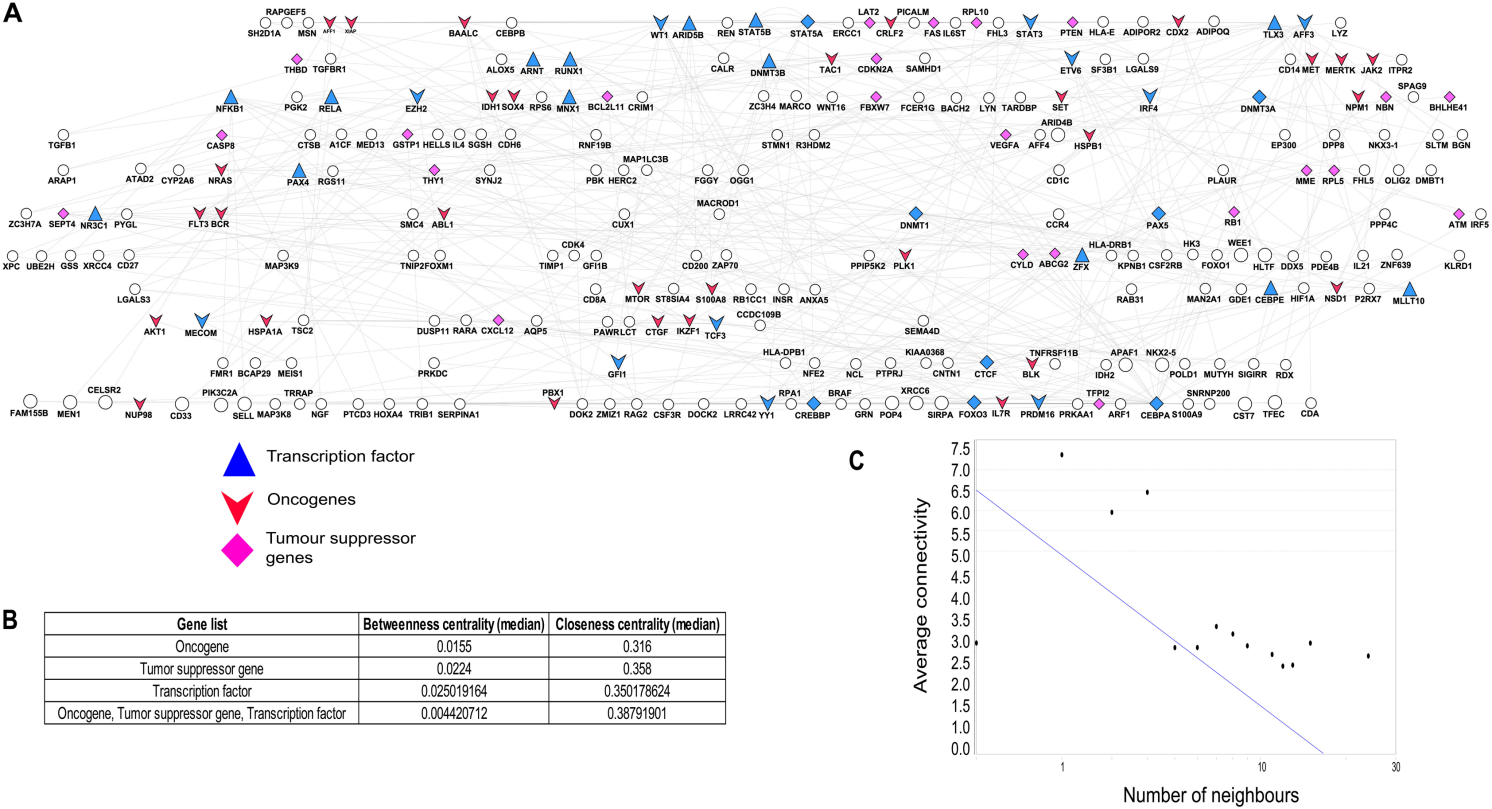
A network perspective of the validated consistent oncogenes and TSGs in childhood ALL. (A) Constructed hierarchical regulatory network of paediatric ALL-related oncogenes, TSGs and transcription factors. The orange triangle node represents the transcription factor genes, the pink V-shape node represents the modulators while the red circle node represents the childhood ALL-related target genes. (B) The results of betweenness and closeness centrality in the regulatory network generated by the NetworkAnalyzer. (C) The plot of in- and out-degree in the regulatory network. In degree shows the number of nodes which instantly connect to and are modulated by the node of interest.

Based on our computational pipeline, we focused on the consistent regulatory motifs and identified a regulatory network in post-translational level. The resulted network from TARGET dataset was shown in Fig. 2A with 176 unique genes, which provide the first insights into the architecture of the paediatric ALL-related genes. The functional enrichment analysis was further conducted based on the 176 unique genes presented in the gene regulatory network. These genes were enriched in several functional classifications related to haematopoiesis. Most of the functional annotations corresponded to the development of leukemia including “regulation of transcription from RNA polymerase II promoter” (GO:0045944, *p*-value = 6.97E−16), “regulation of cell proliferation” (GO:0008284, *p*-value = 1.58E−12) and “hematopoietic stem cell proliferation” (GO:0071425, *p*-value = 0.00957). To further evaluate the significance of the network results of the identified 176 unique genes, we randomly selected twenty genes from the pediatric leukemia-related gene list. The functional results showed that most of the corrected *p*-values for the annotated terms were less than 0.01 (Fig. 2C) which shown significantly different by comparing the twenty random selected gene list (Benjamini–Hochberg value, *p*-values < 0.05). These highly enriched haematopoiesis-related network motifs indicated that our regulatory network was closely related to the progression of leukemia and might be useful in finding the significant gene modules in leukemia.

We further examined the degree distribution to discover the number of connected nodes with the individual node in the network (Fig. 2B). We counted the number of random selected node sets (N) whose average degree, betweenness or closeness based on the *p*-values obtained from the NetworkAnalyzer in Cystoscape. Then we took the average *p*-values of the random samples and compare with the real distribution. By comparing the two topological properties we found that all the *p*-values of betweenness centrality and closeness centrality were <0.05 except the closeness centrality from transcription factor with *p*-value 0.35. These *p*-values indicated that the observed regulatory network elements were unlikely created by chance given their high degree of betweenness and closeness centralities (*p*-values < 0.05).

As shown in Fig. 2C, there was a high correlation between in-degrees from oncogenes and TSGs and their out-degrees related to the corresponding target genes (*p*-value = 2.5E−10). The in-degree shows the number of connections for a targeting node to another node while out-degree means the number of connections that are coming out from a node targeting other nodes. The in-degree here determined as the number of tumour suppressor gene/oncogene nodes that directly connect to and regulate the transcription factor nodes, while the out-degree is the number of target gene nodes which directly connect to and are regulated by the transcription factor node. For example, the in-degree and out-degree for transcription factor *STAT5B* were six and four respectively whereas *STAT5B* was modulated by one oncogene and five TSGs and regulated five target genes. The 100 target genes were then applied to perform functional enrichment analysis and the results indicated that these genes were enriched in “regulating the transcriptional activator activity” such as the RNA polymerase II proximal promoter sequence-specific DNA binding (GO:0001077, *p*-value = 3.5E−5). Our results show that these direct regulation target genes could play an important in cancer progression as they were enriched with the cancer-associated pathways.

In the network , the TFs in the middle layer were connected through the regulatory signals from oncogenes and tumour suppressor genes to the target genes at the bottom layer. However, there were also 39 genes including oncogene, tumour suppressor gene and TF from 176 target genes in our regulatory network. The 39 genes with numerous features established 41 regulatory loops between transcription factors and TSGs/oncogenes (Table S3). We identified two different TFs (*ARID5B* and *RUNX1*) that were regulating the identical target gene (*DOK2*). *ARID5B* gene encodes a member of the AT-rich interaction domain (ARID) family of DNA binding proteins. The genetic alterations ARID5B transcription factor include deletion mutations and are normally involved in the B-cell development (Lahoud et al., 2001). A few studies have reported that *ARID5B* is associated with increased risk of childhood ALL (Evans et al., 2014; Lin et al., 2014). Interestingly, we identified two transcription factors, *STAT5A* and *STAT5B* that formed a reversible regulatory loop. Two loops were formed between *STAT5A* with genes encoding *STAT5B* and *CTGF* while *STAT5B* formed a single regulatory loop with *STAT5A*. In response to the microenvironment signals, *STAT5A* is commonly triggered by the phosphorylation (Wang & Bunting, 2016)and it’s activated via intracellular tyrosine kinases signalling which is the driver of leukemogenesis in hematopoietic cells (Wang & Bunting, 2016). This gene may play a significant role for the survival of progenitor B cells, cell proliferation and differentiation in childhood ALL (Heltemes-Harris et al., 2011). The increased gene expression of the activated *STAT5* has been found to be correlated with poor prognosis in ALL patient cells (Heltemes-Harris et al., 2011). We then further determined the biological process of these 39 target genes and their funtional enrichment results indicate that they were enriched with “regulation of gene expression” (GO:0010629, *p*-value = 5.789E−8), “regulation of cell death” (GO:0010941, *p*-value = 6.841E−8) and “regulation of cell cycle” (GO:0051726, *p*-value = 4.952E−7). As a result, these target genes might play an important role in the development of B-cell in childhood ALL.

### Different biological roles found in oncogenes and TSGs

**Figure 3:**
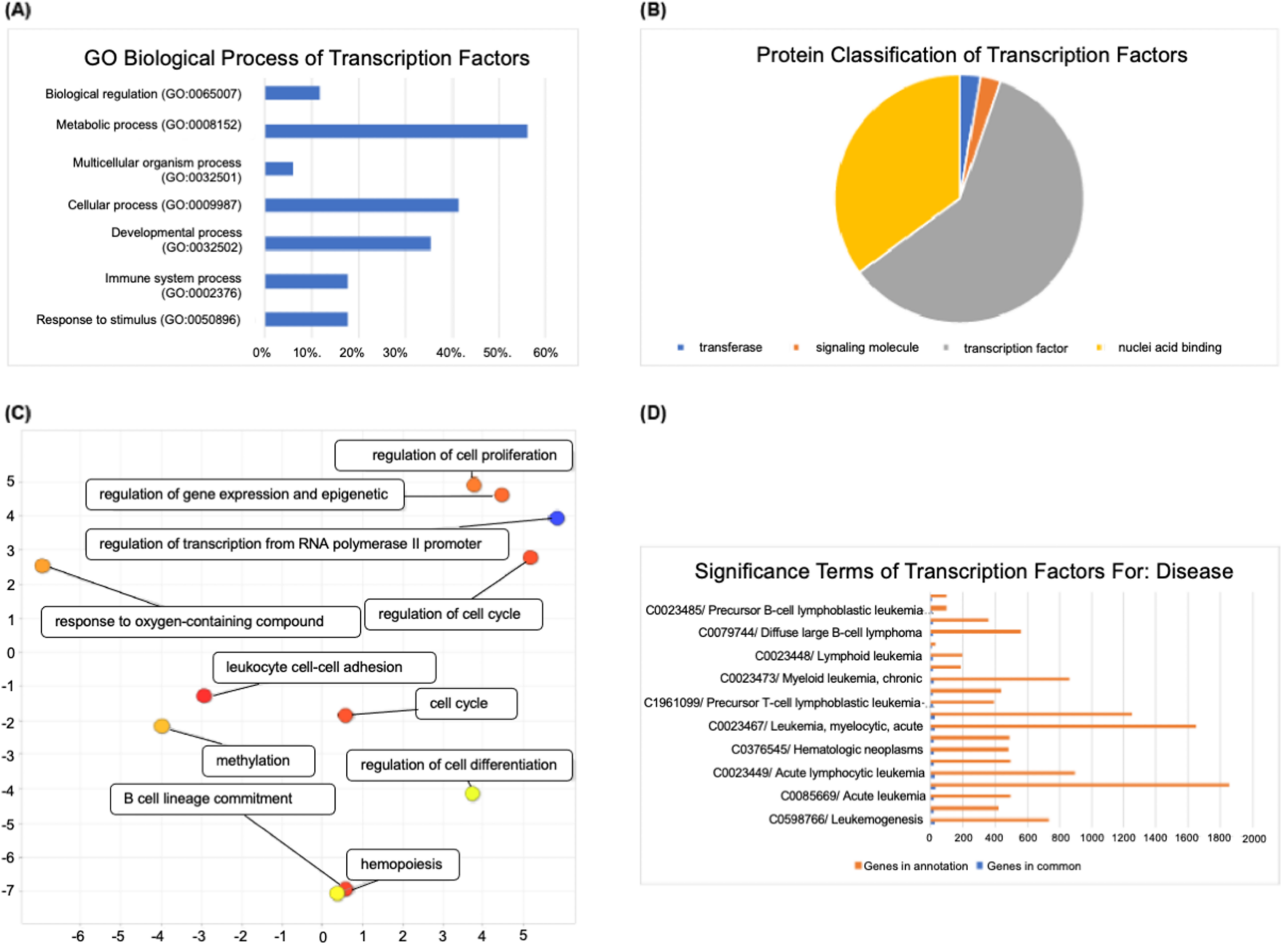
The functional enrichment analysis of the childhood ALL-related transcription factors. (A) The GO biological process of the paediatric ALL-related transcription factors generated by Toppfun analysis. (B) The protein classification of transcription factors generated by PANTHER analysis. (C) The transcription factors enriched with several biological processes generated by REVIGO. (D) The significant terms of transcription factors for certain disease generated by Toppfun analysis.

To further investigate the biological process of the identified oncogenes and TSGs in the constructed regulatory network, we performed an independent functional enrichment analysis particular for the oncogenes and TSGs (Fig. 3). Our results show that these oncogenes and TSGs were associated with diverse biological processes in the leukemia cancer progression. The 40 oncogenes were mostly enriched with positive regulation of gene expression, macromolecule biosynthetic process, cell death and hemopoiesis, whereas the 34 TSGs were mostly involved in metabolic process and negative regulation of cell death, such as *BCL2L1*, an apoptosis suppressor gene which is found in most of the leukemia types, and, opposite to the oncogene results - negative regulation of cell death. Cancer cells likely require a block in apoptosis in order to survive. Overexpression of the anti-apoptotic regulator *BCL-2* gives a block in cell death program which is commonly found in cancer cells (Del Gaizo Moore et al., 2008). The study suggests that *BCL-2* inhibitor can be effective as a single agent in ALL treatment (Del Gaizo Moore et al., 2008).

### Oncogenes and TSGs possessed distinct regulatory pattern

To examine the distinct regulatory pattern of the oncogenes and TSGs in our regulatory network, we created two groups of regulatory profiles for the 74 modulators (40 oncogenes and 34 TSGs). One of the profiles was constructed based on the direct regulation of oncogenes or TSGs for the 34 transcription factors and the other profile was constructed based on the indirect regulation of oncogenes or TSGs for the 176 target genes via transcription factors (see “Materials and Methods”). The hierarchical clustering analyses were separated based on the two profiles: one category included all 40 oncogenes and the other category comprised of 34 TSGs. Overall, these findings implied that oncogenes and TSGs might play roles in leukemia cancer progression that were different from the result which demonstrated that they were incorporated in different biological processes in childhood ALL cancer progression.

Since target genes could influence to the genetic variation in childhood ALL, we explored the biological processes of the oncogenes and TSGs from the clustering results of the target genes. As shown in Table 1, there were two clusters identified in the oncogene and the tumour suppressor gene category. In the first cluster, the 27 genes *(SOX4, NSD1, JAK2, KRAS, NPM1, S100A8, ETV6, KMT2A, MET, MECOM, AFF1, IRF4, AKT1, IKZF1, TCF3, CTGF, CDX2, RUNX1, PBX1, HSPA1A, KIT, PRDM16, YY1, HSPB1, NOTCH1, STAT3)* were annotated with GO functional annotation term “regulation of macromolecule biosynthetic process” (GO:0010557, *p*-value = 3.213E−17). Besides, these 27 oncogenes also associated with “positive regulation of gene expression” (GO:0010628, *p*-value = 1.54E−16). For example, *STAT3* has been reported to be involved in the regulation of gene expression level with JAK/STAT signalling pathway in childhood leukemias (Adamaki et al., 2015). Hence, these 27 oncogenes might possess significant roles in regulating the macromolecule biosynthetic process and gene expression levels. The 25 genes *(WT1, SOX4, NSD1, JAK2, KRAS, NPM1, S100A8, ETV6, KMT2A, MET, MECOM, AFF1, IRF4, AKT1, IKZF1, TCF3, CDX2, RUNX1, PBX1, HSPA1A, KIT, PRDM16, YY1, NOTCH1* and *STAT3*) found in the second cluster were mostly involved in the modulation of transcriptional process as they were enriched with the GO biological term “regulation of nucleic acid-templated transcription” (GO:0051172, *p*-value = 2.14E−18), “regulation of transcription, DNA-templated” (GO:0051253, *p*-value = 3.78E−18) and “regulation of RNA biosynthetic process” (GO:0010558, *p*-value = 5.73E−18). Therefore, these 25 oncogenes from the second cluster were grouped into the same category as they might function in the positive regulation of transcription process.

**Table 1:** The functional annotations (GO biological terms) of oncogenes, TSGs, oncogene-specific target genes and tumour suppressor-specific target genes.

Biological function	*P*-value
**41 oncogenes (modulator)**	
positive regulation of macromolecule biosynthetic process (GO:0010557)	3.21E−17
positive regulation of gene expression (GO:0010628)	1.54E−16
positive regulation of nucleic acid-templated transcription (GO:1903508)	1.80E−16
positive regulation of transcription, DNA-templated (GO:0045893)	1.80E−16
positive regulation of RNA biosynthetic process (GO:1902680)	2.37E−16
**48 TSGs (modulator)**	
negative regulation of nucleobase-containing compound metabolic process (GO:0045934)	1.61E−016
negative regulation of nitrogen compound metabolic process (GO:0051172)	1.23E−015
negative regulation of transcription by RNA polymerase II (GO:0000122)	2.25E−015
negative regulation of gene expression (GO:0010629)	1.23E−014
negative regulation of RNA metabolic process (GO:0051253)	3.49E−014
**19 oncogene-specific target genes**	
regulation of MAPK cascade (GO:0043408)	5.78E−010
MAPK cascade (GO:0000165)	5.51E−009
signal transduction by protein phosphorylation (GO:0023014)	7.79E−009
regulation of cell differentiation (GO:0045595)	8.78E−009
regulation of protein phosphorylation (GO:0001932)	1.85E−008
**25 tumour suppressor-specific target genes**	
regulation of apoptotic process (GO:0042981)	6.57E−011
regulation of programmed cell death (GO:0043067)	7.70E−011
apoptotic process (GO:0006915)	1.30E−010
positive regulation of multicellular organismal process (GO:0051240)	1.50E−010
programmed cell death (GO:0012501)	1.63E−010

In the tumour suppressor gene category, the first cluster (Table 1) included 29 genes (*BHLHE41, CYLD, CDKN2A, FOXO3, NPM1, TCF3, CDX2, CEBPA, RB1, CTCF, NFKB1, WT1, ETV6, NBN, IKZF1, PAX4, PAX5, RUNX1, EZH2, PLK1, CREBBP, NOTCH1, TP53, STAT3, DNMT1, DNMT3A, DNMT3B, FBXW7* and *VEGFA*) that were annotated with GO biological processes “regulation of nucleobase-containing compound metabolic process” (GO:0045934, *p*-value = 2.152E−19) and “regulation of nitrogen compound metabolic process” (GO:0051172, *p*-value = 2.144E−18). In the second cluster, there were 23 genes enriched with “regulation of transcription by RNA polymerase II” (GO:0000122, *p*-value = 1.132E−18). Furthermore, we found that the genes from the third cluster were associated with “regulation of RNA metabolic process” (GO:0051253, *p*-value = 3.775E−18). As a result, the genes in the second and third cluster may possess an important role for the regulation of RNA in transcriptional process. For the genes presented in the fourth cluster, these genes were enriched with “negative regulation of macromolecule biosynthetic process” (GO:0010558, *p*-value = 5.731E−18): the opposite result to the first cluster of oncogenes, which showed positive regulation of macromolecule biosynthetic process. Overall, our findings suggested that oncogenes in ALL development were primarily enriched with positive regulation of macromolecule biosynthetic processes and gene expression, while TSGs were mainly associated with the negative transcriptional regulation of RNA polymerase and negative regulation of macromolecule biosynthetic process.

Interestingly, we observed that the target genes that are regulated by the oncogene and TSGs, demonstrated a competitive regulatory pattern in distinct biological procedures compared to their modulators respectively (oncogenes and TSGs). In the first observation for the oncogene-specific target genes, the genes were enriched with the GO biological process term “regulation of MAPK cascade” (GO:0010629, *p*-value = 1.65E−15) which was different from the GO biological process for the first cluster of modulator oncogene with “regulation of gene expression”. Although the 41 modulator oncogenes were more specific to the regulation of macromolecule biosynthetic process, gene expression and RNA transcription, the oncogene-specific target genes were mainly annotated with regulation of protein phosphorylation (GO:0001932, *p*-value = 1.85E−008) and MAPK cascade (GO:0000165, *p*-value = 5.51E−009). Interestingly, these biological processes were not found in the 48 modulator tumour suppressor gene nor the second cluster in the tumour suppressor category. However, these target genes were also enriched with cell differentiation (GO:0045595, *p*-value = 8.78E−009) which was not observed in any of the clusters in the 40 oncogenes and 34 TSGs lists. In addition, based on the GO annotation, these were also annotated with “signal transduction by protein phosphorylation”. For instance, transforming growth factor beta 1 (*TGFB1*) plays a key role in the TGF-β/SMAD signalling pathway that associates with the natural killer cell immune evasion system in childhood ALL (Rouce et al., 2016). Hence, these oncogene-specific target genes may be related to the regulation of MAPK cascade and cell differentiation in the development of childhood ALL.

In contrast to 25 tumour suppressor-specific target genes were involved in cell death but were not found in any cluster of the 34 modulator tumour suppressor gene category. Overall, these oncogenes and their corresponding specific target genes may play a significant role in MAPK cascade and cell differentiation, whereas the TSGs and their corresponding individual target genes might promote apoptosis. As a result, our regulatory network shows that, although some of the oncogenes were associated with positive regulation, TSGs were mainly involved in the regulation of RNA polymerase II in transcription. However, their individual target genes suggest an opposite biological function and that oncogene-specific target genes may be involved in cell differentiation and tumour suppressor-specific target genes were mostly enriched with apoptosis.

**Figure 4:**
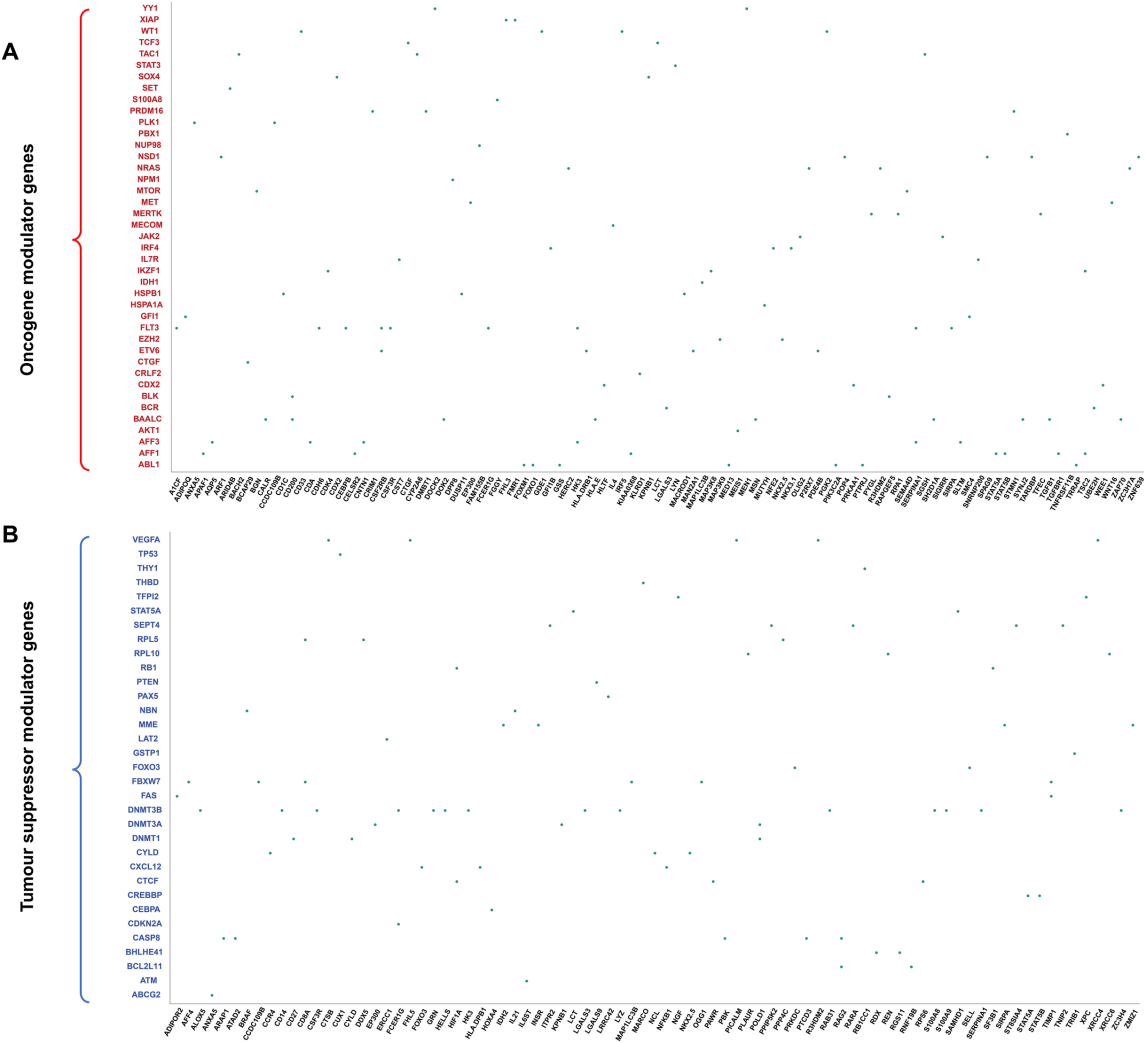
Downstream target gene profiling with oncogenes and TSGs from the consistent motifs. The dot plot illustrates the regulatory relationship identified between the modulators (oncogenes and TSGs) and their respective target genes. (A) Oncogene modulator genes; (B) TSG modulator gene.

The dot plot in Fig. 4 represented the regulatory relationship between modulator genes and their corresponding downstream target genes. The modulators’ dendrogram is divided into two main branches shown in the dot plot (A—oncogene modulator genes; B—Tumour suppressor modulator genes). The first oncogene branch as an example: the enriched genes are involved in “positive regulation of gene expression” while in the second TSGs branch, the genes were mainly involved in the “negative regulation of gene expression”. These observations indicated that oncogenes and TSGs might play different roles in childhood ALL progression, which is consistent with the above investigation that they were involved in distinct biological functions.

### Interplay of oncogenes, TSGs and their corresponding modulating TFs to regulate cell death, cell proliferation, haematopoiesis and activation of leucocytes and lymphocytes

**Figure 5:**
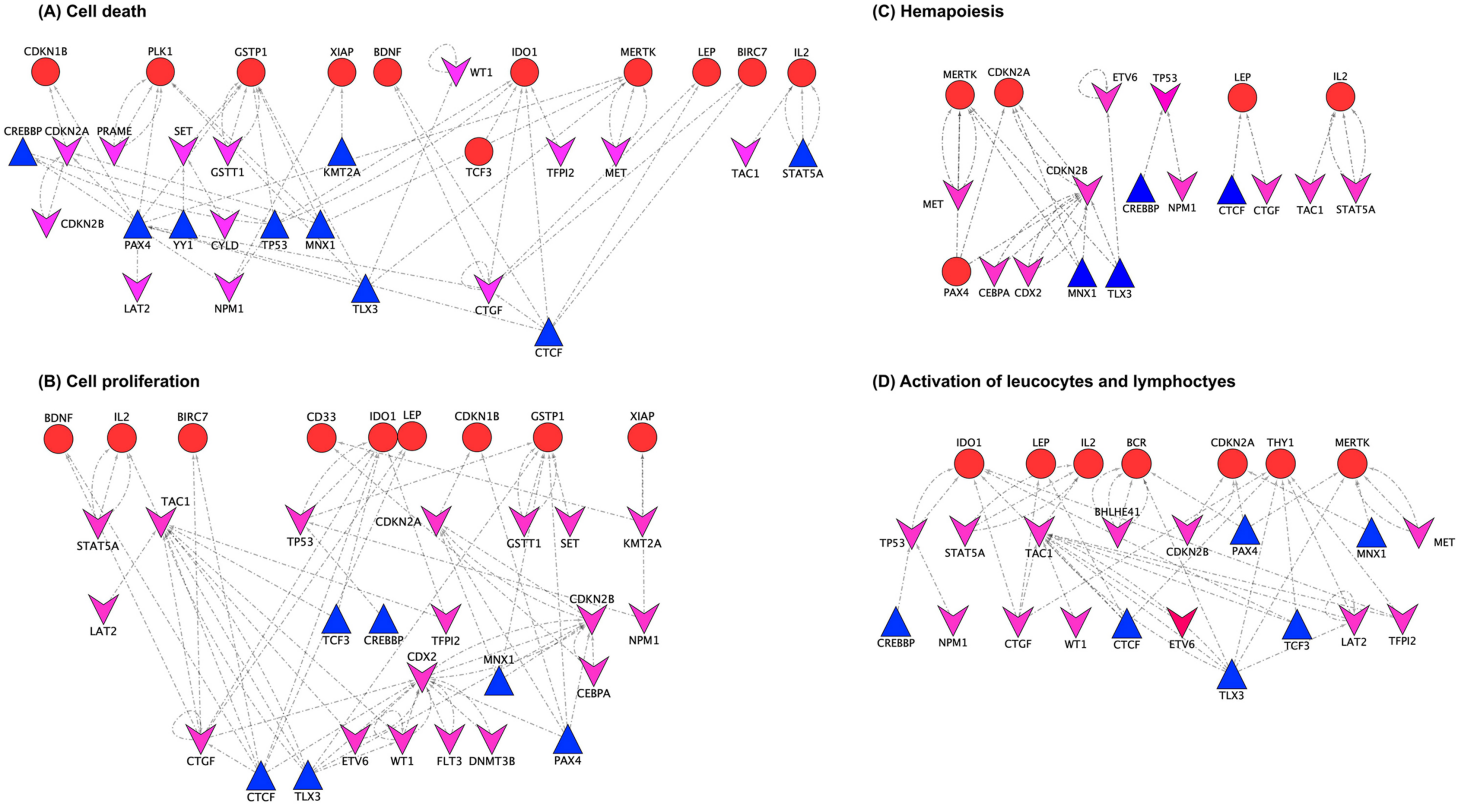
Biological interaction of oncogenes and TSGs to modulate the four different biological processes. (A) Cell death. (B) Regulation of cell proliferation. (C) Response to haematopoiesis. (D) Activation of leucocytes and lymphocytes.

To better understand the gene regulation of the significant biological activities involved in the childhood ALL cancer progression, we analysed and created four different subnetworks related to “cell death”, “cell proliferation”, “haematopoiesis” and “activation of leucocyte and lymphocyte”. To construct particular networks, we identified those transcription factors that directly regulated the target genes in each individual biological process by filling the regulatory gaps between the top layer (oncogenes/TSGs) and the bottom layer (target genes). In the cell death network, there are seven oncogenes, eight TSGs, nine transcription factors and 11 target genes. Figure 5 shows all the transcription factors were directly regulating the target genes in the cell death subnetwork. These transcription factors have been reported to be involved in other cancer types: *TP53* (Fridman & Lowe, 2003; Ozaki & Nakagawara, 2011), *PAX4* (Hata et al., 2008), *CTCF* (Docquier et al., 2005; Venkatraman & Klenova, 2015), *MNX1* (Lv et al., 2017), *TLX3* (Dadi et al., 2012), *STAT5A* (Du et al., 2012) and *CREBBP* (Tang et al., 2016). Remarkably, *STAT5A* is involved in the induction of tumour cell apoptosis in leukemia cells (Kaymaz et al., 2013). Likewise, five transcription factors were demonstrated to play a key role in cell proliferation namely *PAX4* (Lorenzo et al., 2015), *TLX3* (Renou et al., 2017), *MNX1* (Lv et al., 2017), *TCF3* (Taniue et al., 2016) and *CTCF* (Lee et al., 2017). Notably, *TLX3* induces cell proliferation of T-cell in leukemia (Van Vlierberghe & Ferrando, 2012). In the subnetwork of hemopoiesis, four transcription factors - *TLX3* (Sive & Gottgens, 2014; Van Vlierberghe & Ferrando, 2012), *MNX1* (Wildenhain et al., 2012), *CTCF* (Torrano et al., 2005) and *CREBBP* (Goyama & Kitamura, 2017; Kung et al., 2000) have been shown in previous experimental studies to be associated with hemopoiesis. In addition to hemopoiesis, these four transcription factors were found to be associated with activation of leucocyte and lymphocyte in the fourth subnetwork as shown in Fig. 5. For instance, *TCF3* that found in the fourth subnetwork was reported to take part in the regulation of lymphocytes (Andrysik et al., 2013). Overall, among the 17 transcriptions factors presented in all these four subnetworks, according to the literature, 15 transcription factors support the novel biological processes in their respective subnetworks.

Our results indicated that most of the oncogenes and TSGs interplayed in the biological interactions, and they are likely to impact regulation to each other in a given cellular process. In the cell death subnetwork, 40% of oncogenes, 70% of TSGs and 80% of transcription factors shared the common genes in cell proliferation subnetwork (Fig. S1). In addition, 15 common genes among the 29 were found to be shared between haematopoiesis and the activation of leucocyte and the lymphocyte subnetwork. Hence, oncogenes and TSGs might co-regulate the corresponding cellular mechanisms in opposite ways, including cell death and cell proliferation. Both oncogenes and TSGs may have significant function in the regulation of haematopoiesis and activation of leucocyte and lymphocyte.

## Discussion

In this study, we investigated the gene regulatory networks of the childhood ALL involving oncogenes, TSGs, and TFs by incorporating the gene expression profiles. Instead of focusing at the transcriptional level, we constructed a genetic regulatory network to compare gene regulation at the post-translational level. Our computational framework started with the extraction of the modulating genes (oncogenes and TSGs) and TFs, and we then inferred regulatory relationships using gene expression profiles. Our aim was to determine whether distinct regulatory patterns exist between oncogenes and tumour suppressor genes in childhood ALL. To this end, we analysed and constructed the hierarchical regulatory networks by incorporating the gene expression data from the TARGET database and GEO database , and we explored the modulating effects of oncogenes and tumour suppressor genes at the post-translational level. Based on these relationships, we constructed a three-layer regulatory network that included oncogenes, TSGs, TFs, and their joint target genes. This overview network analysis framework is likely robust to identify some important genes and their regulation in childhood ALL cancer progression. The result may provide hints to inferring the gene expression regulatory networks with significant biological processes involved in childhood leukemias.

By comparing two independent gene expression data sets (TARGET and GEO), we were able to identify the distinct regulatory patterns between oncogenes and TSGs, which may provide the fundamental information about post-translational gene regulation during cancer development. We found that the oncogenes were mainly enriched with positive regulation and TSGs were mainly involved in the regulation of RNA polymerase II in transcription. However, their individual target genes suggest an opposite biological function and that oncogene-specific target genes may be involved in cell differentiation and tumour suppressor-specific target genes were mostly enriched with apoptosis. Therefore, different regulation mechanism was identified between TSGs and OCGs in our analysis, which suggested that these modulator genes could play an important role during cancer development in childhood ALL.

Many common leukemia causal genes, such as *BAALC*, *TP53* and *STAT* was ranked at the top in our gene list. Hence, our gene list which identified from our genetic regulatory study together with the used datasets, might be served as a valuable resource for the leukemia research community to further examine the cancer progression in childhood ALL. On top of that, the ALL-specific regulatory network that constructed in this study might hold the key to understanding the regulatory mechanisms between the highly connected TFs in the network, their modulators, and novel biological functions of TFs; subsequently, these relationships could provide some interesting clues for further examination. For example, the genetic looping interactions are known to be associated with the expression of *ARID5B* (Studd et al., 2017). *ARID5B* may serve as a transcriptional activator that enhances the regulatory network triggered by the *TAL1* complex in T-ALL cell proliferation (Tan, Zhang & Sanda, 2019). In recent years, studies showed that the mutations and single nucleotide polymorphisms of TF, *ARID5B* are involved in the oncogenesis of ALL. Ge Zheng et al. reported that the overexpression of *ARID5B* is associated to leukemic cell proliferation and several poor prognostic markers (Ge et al., 2018). In addition, previous GWAS studies (Shen, Chen & Lacorazza, 2017; Trevino et al., 2009) have reported that *ARID5B* with the frequent polymorphism involved in lymphoid differentiation that will increase the risk of childhood ALL.

Until now, based on our knowledge, there has been no report of the cross-validation analysis of gene regulation model in childhood ALL. With a better understanding of the genetic elements including their associated genetic pathways in childhood ALL that induce cancer susceptibility, the genetic regulatory analysis could help to accelerate the development of new targeted therapies. We hope the data presented in this study will represent as a valuable resource for the paediatric ALL research community to explore both oncogenes and TSGs in childhood ALL carcinogenesis processes.

## Conclusions

In summary, we present a computational strategy to establish a hierarchical regulatory network from TARGET and GEO childhood ALL gene expression data with TFs as bridges to connect the significant modulators (oncogene and TSGs) to their corresponding potential targets. The cross-validation analysis has identified 205 consistent regulatory motifs by comparing the regulatory results of TARGET with three different GEO data sets. The childhood ALL-regulatory network result generated in this study might be composed of the significant regulatory relationships between hub TFs (highly connected TFs in the network), their modulators, and novel functions of TFs in the childhood ALL cancer progression. Eventually, these information about these relationships could provide useful and interesting clues for further investigation. Our results provide a unique opportunity to fill gaps in knowledge about the role of oncogenes and tumour suppressor genes in the gene-regulation at post-translational level by performing a cross-validation analysis using different gene expression profiles.

## Supplemental Information

10.7717/peerj.11803/supp-1 Supplemental Information 1 Common genes in the four hierarchical subnetworks—cell death (CD), cell proliferation (CP), haematopoiesis (HP) and leucocyte and lymphocyte activation (LLA).

10.7717/peerj.11803/supp-2 Supplemental Information 2 The Cytoscape session file for the network perspective of the validated consistent oncogenes and TSGs in childhood ALL in Figure 2.

10.7717/peerj.11803/supp-3 Supplemental Information 3 The Cytoscape session file for the cell death subnetwork in childhood ALL (Figure 5A).

10.7717/peerj.11803/supp-4 Supplemental Information 4 The Cytoscape session file for the cell proliferation subnetwork in childhood ALL (Figure 5B).

10.7717/peerj.11803/supp-5 Supplemental Information 5 The Cytoscape session file for the hemopoiesis subnetwork in childhood ALL (Figure 5C).

10.7717/peerj.11803/supp-6 Supplemental Information 6 The Cytoscape session file for the leukocyte lymphocyte activation subnetwork in childhood ALL (Figure 5D).

10.7717/peerj.11803/supp-7 Supplemental Information 7 The gene list of 205 validated consistent motifs from TARGET and GEO.

10.7717/peerj.11803/supp-8 Supplemental Information 8 Gene list of four subnetworks on cell death, cell proliferation, haematopoiesis and activation of leucocytes and lymphocytes.

10.7717/peerj.11803/supp-9 Supplemental Information 9 List of regulatory loops in the childhood ALL-specific regulatory network.

## Additional Information and Declarations

### Data Availability

The following information was supplied regarding data availability: The raw data used were from public genomics databases: GSE17703, GSE43209 and GSE72623 and TARGET.
