# Supplementary figures and images for: Identification of consistent post-translational regulatory triplets related to oncogenic and tumour suppressive modulators in childhood acute lymphoblastic leukemia
Source: PeerJ. 2021 Jul 14;9:e11803. doi: 10.7717/peerj.11803 (PMC8286060; doi:10.7717/peerj.11803)

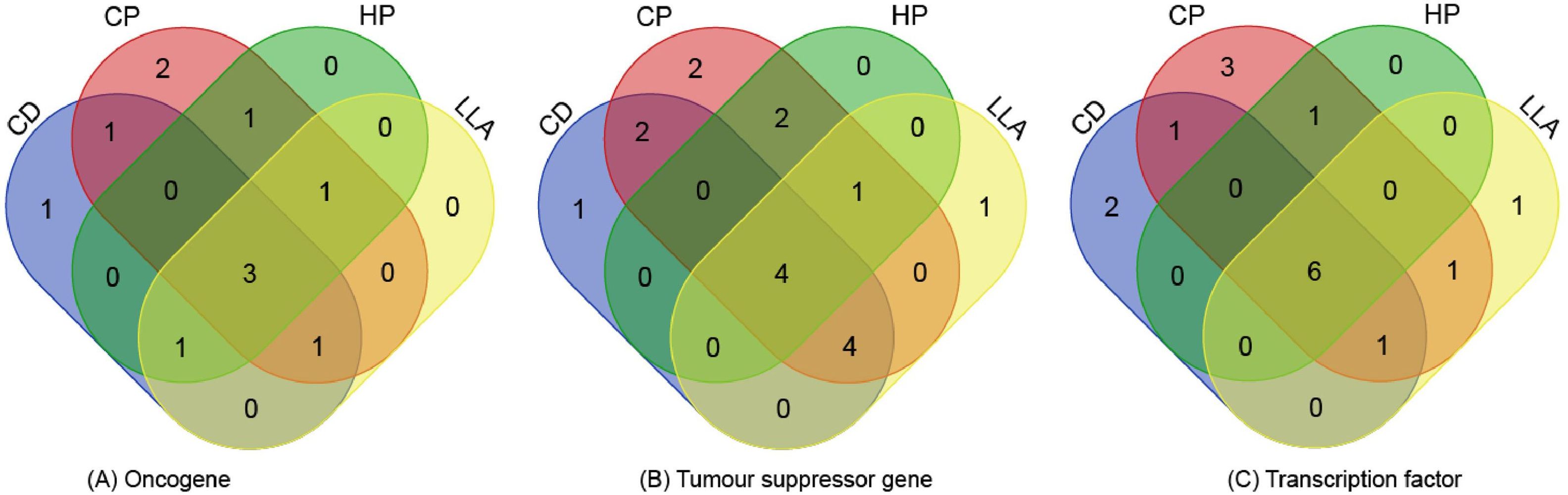

Supplement: Supplemental Information 1 [file peerj-09-11803-s001.jpg]
